# The effect of the attitude towards risk/ambiguity on examination grades: cross-sectional study in a Portuguese medical school

**DOI:** 10.1007/s10459-023-10305-z

**Published:** 2024-01-15

**Authors:** Filipe Leite-Mendes, Luis Delgado, Amelia Ferreira, Milton Severo

**Affiliations:** 1 Faculty of Medicine, University of Porto, Rua Hernani Torres, 247, 8º DRT FRT. 4200-320, Porto, Portugal; 2 Department of Pathology, Basic and Clinical Immunology, Faculty of Medicine, University of Porto, Porto, Portugal; 3 Immunoallergology Unit, University Hospital Centre of São João, Porto, Portugal; 4 Epidemiology Research Unit, Institute of Public Health (EPIUnit), University of Porto, Porto, Portugal; 5 ICBAS - School of Medicine and Biomedical Sciences, University of Porto, Porto, Portugal

**Keywords:** Ambiguity aversion in medicine, Attitude towards risk, Formula scoring, Knowledge, Medical students, Multiple choice examination, Multiple choice questions, Negative marking, Number-right scoring, Personality, Reliability, Tolerance for ambiguity, Validity

## Abstract

Given the high prevalence of multiple-choice examinations with formula scoring in medical training, several studies have tried to identify other factors in addition to the degree of knowledge of students which influence their response patterns. This study aims to measure the effect of students’ attitude towards risk and ambiguity on their number of correct, wrong, and blank answers. In October 2018, 233 3rd year medical students from the Faculty of Medicine of the University of Porto, in Porto, Portugal, completed a questionnaire which assessed the student’s attitudes towards risk and ambiguity, and aversion to ambiguity in medicine. Simple and multiple regression models and the respective regression coefficients were used to measure the association between the students’ attitudes, and their answers in two examinations that they had taken in June 2018. Having an intermediate level of ambiguity aversion in medicine (as opposed to a very high or low level) was associated with a significant increase in the number of correct answers and decrease in the number of blank answers in the first examination. In the second examination, high levels of ambiguity aversion in medicine were associated with a decrease in the number of wrong answers. Attitude towards risk, tolerance for ambiguity, and gender did not show significant association with the number of correct, wrong, and blank answers for either examination. Students’ ambiguity aversion in medicine is correlated with their performance in multiple-choice examinations with negative marking. Therefore, it is suggested the planning and implementation of counselling sessions with medical students regarding the possible impact of ambiguity aversion on their performance in multiple-choice questions with negative marking.

**Supplementary Information:**

The online version contains supplementary material available at 10.1007/s10459-023-10305-z.

## Introduction

In the assessment of medical students, multiple-choice examinations (MCEs) are often used. The two most used scoring methods are the number-right scoring and the formula scoring (Muijtjens et al., 1999; Ndu et al., 2016). The score on the first method corresponds to the sum of the correct answers. With formula scoring, in addition to the correct answers (C), the final score (S) takes into account the number of wrong answers (W) and the number of distractors (D, number of incorrect options), according to the following formula “S = C − (W/D)” (Severo et al., 2015).

The use of formula scoring has the primary objective of countering the effect of answering randomly potentially present in the number-right scoring method: avoiding the introduction of random noise, seeking to account only for the true knowledge of the student. This method increases (at least in theory) the validity and reliability of the test (Muijtjens et al., 1999; Severo et al., 2015). A second objective of this system is to encourage the student to recognize what he does not know, giving him the option in all questions not to answer (leaving them “blank”), or to answer “I don't know”. This attitude, considered desirable in future clinical practice, is not cultivated by MCEs with number-right scoring, where the response rate is usually very close to 100%, regardless of the student's level of knowledge (Ndu et al., 2016).

In line with theoretical predictions, empirical research in the area has shown that reliability is preserved or increased in MCEs with formula scoring in face of number-right scoring. That is, there is an equal or smaller probability of two students with the same knowledge obtaining ratings different due to luck or randomness (Severo et al., 2015). However, Muijtjens et al. (1999) point out as a disadvantage of the formula scoring method the underestimation of students' knowledge and, therefore, less validity: students tend to leave blank answers to questions for which they have partial knowledge, i.e., for which they would have a higher probability than chance of getting it right.

Regarding the effects on the classification in MCEs, the work of Harden et al. (1976) (Goldik, 2008) revealed that the use of formula scoring produced lower classifications compared to the number-right scoring, although the students' ranking was maintained. In the work of Severo et al. (2015), the reliability was identical in both methods, however, there was a lower approval when formula scoring was used.

Given the prevalence of this evaluation method in medical training, several studies have sought to identify other factors, in addition to the degree of knowledge of students, that influence the response patterns in MCEs, thereby decreasing its validity and reliability. The contradictory results regarding gender are highlighted: on the one hand, there seems to be evidence of negative discrimination against women, who tend to risk less (although they make better use of the options, compensating for the final score) (McG Harden et al., 1976); on the other hand, there are studies in which gender has no effect on student response patterns (Ndu et al., 2016). Another variable that seems to influence responses in MCEs with formula scoring is the type of instructions provided about the test: when the examiners verbally emphasize or write in the statement “the penalty for a wrong answer is severe”, there is a clear tendency to leave blank answers (McG Harden et al., 1976). The role of attitude towards risk—understood in this work as one of the determinants of risk-taking behaviour (Nosic, 2010)—has also been studied in this context: comparing two students with the same degree of knowledge, Muijtjens et al. (1999) found that the one most willing to risk will, on average, have fewer blank responses, as well as more satisfactory results; the authors therefore conclude that the formula scoring method is penalizing for students less likely to take risks. However, these results are not seen in the work of Ndu et al. (2016), where the attitude towards risk had no effect on the response patterns and classifications of students in MCEs with formula scoring.

Research regarding the effect of personality in the responses to MCEs with formula scoring has a long history, going at least as far back as 1954 when Sherriffs et al. (1954) found that students with high scores in the A scale of the MMPI, which are characterized by introversion, rumination, anxiety, low self-esteem, and undue concern with the impression they make on others, are handicapped by being scored using formula scoring. This effect remained even when the students' knowledge of course material is held constant, and suggests that these students are penalized by their tendency to omit more items, and to omit items the answers to which they know. The debate for and against formula scoring has an even longer history, but recently several institutions have elected to abandon its use, most notably, Educational Testing Service (ETS), the world's largest private non-profit educational testing and assessment organization. As such, since 2016, the SAT uses number right scoring instead of formula scoring (SAT, 2022).

Another possible source of uncertainty besides the risk or probability of a phenomenon taking place, is ambiguity (the lack of reliability, credibility, or adequacy of information about the phenomenon). Ambiguity has been shown to promote pessimistic appraisals of risk and avoidance of decision making (Han et al., 2011). It could be plausible to assume that those with a lower tolerance for ambiguity, and specifically those with a higher aversion to ambiguity in medicine would be less proficient answering complex medical questions with multiple interpretations and would therefore leave more answers blank and score lower in MCEs with formula scoring.

In the Faculty of Medicine of the University of Porto, there are no common guidelines on how examinations should be made. Some course units/disciplines use formula scoring, others use number right scorings, some allow students to choose more than one answer in multiple-choice questions (MCQs), some also have written sections, and whilst in some there is only one distractor (like true or false questions), in others there are more than 10 distractors.

This study aims to assess the effect of student’s attitude towards risk and ambiguity, in the number of correct, wrong, and blank answers in MCEs with formula scoring.

## Methods

### Study design and participants

This was a cross-sectional study of medical students at Faculty of Medicine of the University of Porto (FMUP), in Porto, Portugal, in 2018. From a total of 268 students enrolled on the 3rd year of the Integrated Master of Medicine (MIMed) at FMUP in the academic year 2018/2019, 233 (86.9%) completed a paper questionnaire which assessed their tolerance of different sources of uncertainty, in October 2018. We recruited students by approaching them after classes. They were informed that participating in this study would be voluntary and that refusing to participate would not result in any disadvantages. Non-respondents were given a second opportunity to complete the questionnaire. From the students enrolled on the 3rd year of (MIMed) 172 (64.1%) had performed a Basic Immunology exam and 157 (58.5%) a Medical Microbiology I exam, in June 2018, when they were in their second academic year of MIMed.

Of the sample of students that took the Immunology exam, 111 out of 172 (64.5%) were female, the average age was 20.4 (SD = 1.49) years, and the course grade average and admission grade were 13.95 (1.37) and 18.72 (0.68), out of 20, respectively.

Of the sample of students that took the Microbiology exam, 107 out of 157 (68.6%) were female, the average age was 20.7 (2.17) years, and the course grade average and admission grade were 13.8 (1.44) and 18.6 (0.79), out of 20, respectively.

### Questionnaire

The questionnaire was distributed on paper to all classes of the 3rd year of MIMed and each student filled in their own questionnaire by hand. Section I included socio-demographic questions (gender and age), number of overdue course units, course grade average (the sum of the grade in each course unit multiplied by its credits, divided by total number of credits for all course units completed by the time they filled in the questionnaire), admission grade (their grade when they entered medical school), and one question about habits of leaving blank answers in MCEs with formula scoring (answer on a Likert scale from 1—"never" to 5—"always"). **S**ections 2, 3 and 4, assessed the student’s risk attitude, ambiguity aversion in medicine and tolerance for ambiguity, respectively.

Section 2 consisted of a *(Pearson) Risk Attitude* (PRA) scale. The original scale by Pearson et al. (1995) has an acceptable reliably (α = 0.71). In this study the Cronbach’s Alpha was 0.79 (CI 95% = 0.74; 0.83), and the percentage explained by first component of each scale was 49%. Students with a higher score in this scale had a greater predisposition to take risks and hence a higher tolerance of risk.

The Section 3 consisted of an *Ambiguity Aversion in Medicine* (AA-Med) scale. The original scale by Han et al. (2009) demonstrated acceptable reliability (α = 73). In this study the α was 0.69 (0.62; 0.75), and the percentage explained by first component of each scale was 45%. Respondents with a higher score in this scale had less interest in a hypothetical ambiguous cancer screening test.

The Section IV consisted of a *Tolerance for Ambiguity* (TFA) scale. The scale by Geller et al. (1990) also has an acceptable reliability (α = 0.75). In this study the α was 0.70 (0.63; 0.76), and the percentage explained by first component of each scale was 38%. Since this scale was negatively scored, respondents with a higher score are said to have a lower tolerance for ambiguity.

All scales were translated to Portuguese and composed of six or seven questions on a six-point Likert scale, from “totally disagree” (one point) to "totally agree" (six points). Therefore, the lowest possible score was six or seven, and the highest was 36 or 42, depending on the scale. The translation process was as follows:

1. Forward translation: translators independently conducted forward translations.
2. Synthesis: the drafts of each translator were compared item by item, and the translations were synthesized.
3. Back-translation: they were then translated back into English by translators who had no prior knowledge of the scale. The back-translated version was compared with the original English version and proofread.
4. Expert review: we asked a medical education expert to review and, thereafter, modified it based on the feedback.
5. Pre-testing: we performed a pilot test with four medical students, who were interviewed on whether the scales exhibited expressional clarity and meaning. Since pilot testing showed no problematic items, the translation process was confirmed.

The survey can be seen in online Appendix A, and extra information about the reliability of each scale can be seen in online Appendix B.

### Examinations

Two different written examinations were used as data to evaluate effect of the three several scales on the students’ response pattern.

The first one was the Basic Immunology examination, which comprised of 100 true/false questions with formula scoring, 95 MCQs rated using the number-right scoring method, some of which were worth more points than each true/false question, and 2 open essay questions.

The second one was the Medical Microbiology I examination, which comprised of 100 true/false questions with formula scoring, and 25 MCQs without formula scoring, which were rated six times higher.

In this study, we only worked with MCQs with formula scoring, which were all true/false questions. All examinations and questionnaires were read with optical reading.

### Statistical analysis

In order to characterize the sample and the questions with formula scoring in both examinations, the statistical analysis carried out included measures of central tendency and dispersion (means and respective standard deviations; medians and respective percentiles). The examinations items difficulty and discrimination index, reliability (using Cronbach's alpha), and standard error of measurement were calculated.

Simple and multiple regression models and the respective regression coefficients were used to measure the association between the scales and the students’ answers. Non-linear/quadratic models are shown when results approached or reached statistically significant quadratic association (α = 0,05), otherwise linear regression models are shown. Model 1 showed crude associations, with no adjustments. Model 2 was adjusted for age, gender, course grade average and admission grade. Model 3 was adjusted to the previous factors as well as for the scores in each of the other scales. A log-transformation was applied when the outcome variable showed a skewed distribution.

All statistical analysis was performed using R Statistical Software (R version 4.0.3, 2020-10-10).

## Results

The characteristics of True/False questions of the Immunology and Microbiology examinations can be seen in (Table 1). Both examinations had good reliability, however the Immunology examination had a greater difficulty index (easier questions and higher scores), greater discrimination index (was better at distinguishing between students who obtained a high and a low final classification), even greater reliability, and lower standard error of measurement.

**Table 1:** Characteristics of True/False Questions

	Immunology mean (SD)	Microbiology mean (SD)
Difficulty index	0.43 (0.20)	0.21 (0.18)
Discrimination index mean (SD)	0.29 (0.10)	0.18 (0.12)
Average score*	42.67 (19.84)	21.27 (16.56)
Number of correct answers mean (SD)	52.60 (19.09)	42.54 (16.62)
Number of wrong answers mean (SD)	9.92 (7.60)	21.27 (12.87)
Number of blank answers mean (SD)	37.48 (21.22)	36.18 (24.68)
	Alpha (IC95%)	Alpha (IC95%)
Cronbach’s Alpha	0.90 (0.88; 0.92)	0.80 (0.76; 0.84)
Standard error of measurement	6.27%	7.40%

*Average Score using formula scoring, out of 100

### Multiple regression models 1 and 2

The PRA and TFA scales did not shown any crude or adjusted association with the number of correct, wrong, and blank answers for either examination (Table 2).

**Table 2:** Linear or quadratic association of PRA, AA-Med and TFA scores with correct, wrong, and blank answers for Immunology and Microbiology examinations

	Immunology			Microbiology		
Outcome:	β	CI 95%	P	β	CI 95%	p
**Exposition: PRA**						
**Correct**						
Model 1	0.06	(− 0.53; 0.64)	0.85	− 0.19	(− 0.71; 0.33)	0.48
Model 2	0.24	(− 0.24; 0.72)	0.32	− 0.03	(− 0.55; 0.49)	0.92
**Wrong**						
Model 1	0.01	(− 0.01; 0.03)	0.26	− 0.21	(− 0.61; 0.19)	0.30
Model 2	0.01	(− 0.01; 0.04)	0.23	− 0.34	(− 0.76; 0.09)	0.12
**Blank**						
Model 1	− 0.24	(− 0.89; 0.41)	0.46	0.40	(− 0.37; 1.17)	0.31
Model 2	− 0.42	(− 1.04; 0.19)	0.17	0.36	(− 0.46; 1.19)	0.39
**Exposition: AA-Med**						
**Correct**						
Model 1						
Linear term	**9.64**	**(4.38; 14.89)**	**< 0.001**	4.49	(− 0.50; 9.49)	0.078
Quadratic term	**− 0.24**	**(− 0.38; − 0.11)**	**< 0.001**	− 0.12	(− 0.24; 0.01)	0.071
Model 2						
Linear term	**5.93**	**(1.75; 10.11)**	**0.006**	2.88	(− 1.99; 7.76)	0.244
Quadratic term	**− 0.15**	**(− 0.26; − 0.046)**	**0.005**	− 0.08	(− 0.20;0.05)	0.212
**Wrong**						
Model 1						
Linear term	0.13	(− 0.07; 0.34)	0.200	− 0.40	(− 0.92; 0.12)	0.13
Quadratic term	− 0.004	(− 0.01; 0.0015)	0.161	–	–	–
Model 2						
Linear term	0.18	− 0.03; 0.39)	0.092	− 0.34	(− 0.87; 0.20)	0.21
Quadratic term	− 0.005	(− 0.01; 0.004)	0.073	–	–	–
**Blank**						
Model 1						
Linear term	**− 10.26**	**(− 16.12; − 4.41)**	** < 0.001**	0.47	(− 0.52; 1.46)	0.35
Quadratic term	**0.26**	**(0.11; 0.41)**	** < 0.001**	–	–	–
Model 2						
Linear term	**− 7.21**	**(− 12.64; − 1.77)**	**0.010**	0.52	(− 0.51; 1.55)	0.32
Quadratic term	**0.19**	**(0.049; 0.32)**	**0.008**	–	–	–
**Exposition: TFA**						
**Correct**						
Model 1	0.29	(− 0.26; 0.84)	0.30	0.13	(− 0.36; 0.63)	0.60
Model 2	0.20	(− 0.23; 0.62)	0.36	− 0.04	(− 0.53; 0.46)	0.89
**Wrong**						
Model 1	0.00	(− 0.02; 0.02)	0.80	0.27	(− 0.11; 0.66)	0.16
Model 2	− 0.00	(− 0.02; 0.02)	0.83	0.25	(− 0.16; 0.65)	0.23
**Blank**						
Model 1	− 0.34	(− 0.95; 0.27)	0.28	− 0.41	(− 1.14; 0.33)	0.28
Model 2	− 0.26	(− 0.81; 0.28)	0.34	− 0.21	(− 0.99; 0.57)	0.59

Model 1: crude model. Model 2: adjusted for age, gender, course grade average, admission grade

Pearson risk attitude (PRA), ambiguity aversion in medicine (AA-Med), and of Tolerance for ambiguity (TFA), respectively

Items in **bold** reached statistical significance

**Fig. 1:**
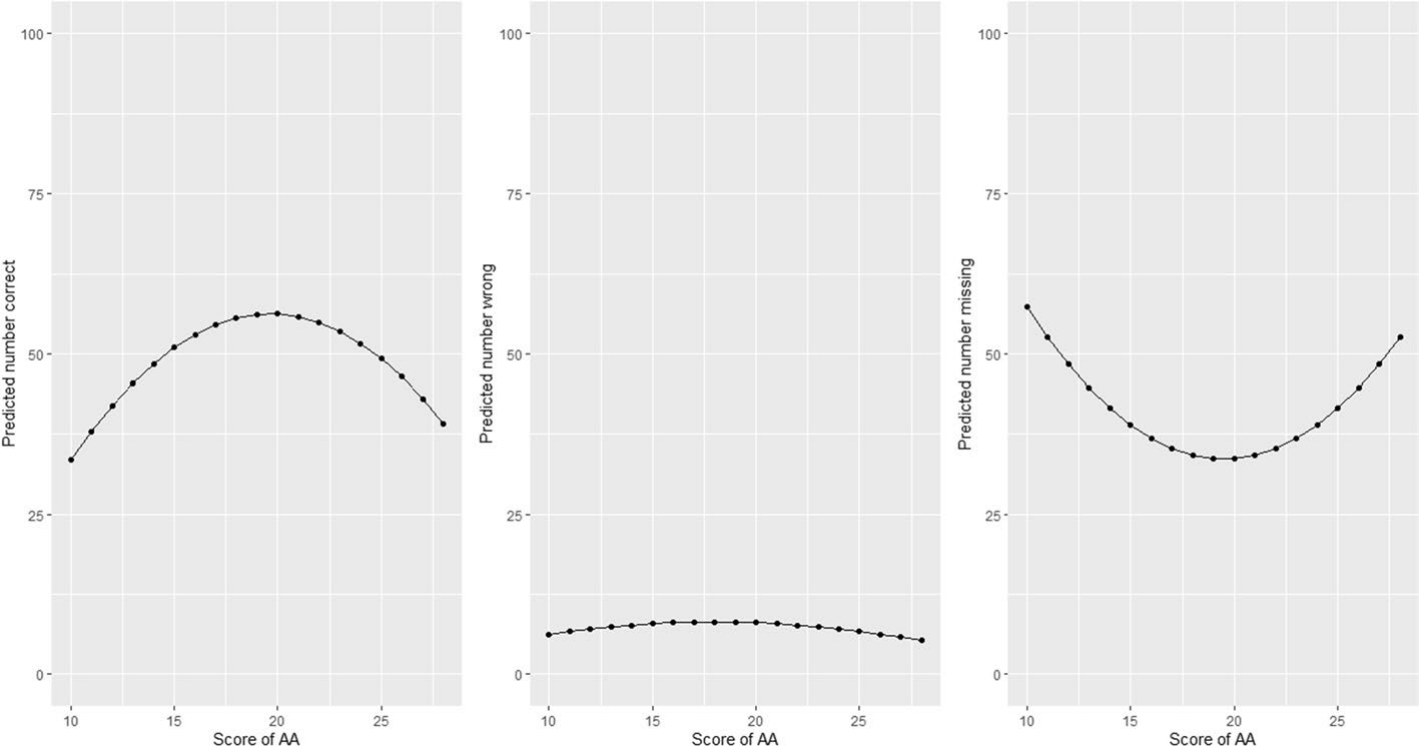
Model 1 AA-Med in immunology exam

The AA-Med scale showed quadratic crude association and even after adjustment for the other factors the association remained with the number of correct and blank answers, and almost significant quadratic adjusted association with the number of wrong answers for the Immunology examination (Table 2 and Fig. 1). Meaning that having an intermediate score in the AA-Med scale (as opposed to a very high or low score) was associated with a statistically significant increase in the number of correct answers and decrease in the number of blank answers in the Immunology examination. The Microbiology examination only showed in crude almost significant quadratic association with the correct answers (Table 2).

### Multiple regression model 3

When the results were adjusted not only for Age, Gender, Course Grade Average, and Admission Grade, as previously shown, but also for each of the scales, the PRA and TFA scales still did not shown any crude or adjusted association with the number of correct, wrong, and blank answers for either examination (Table 3).

**Table 3:** Linear or quadratic association of PRA, AA-Med and TFA scores adjusted between then with correct, wrong, and blank answers for Immunology and Microbiology examinations

	Immunology			Microbiology		
	β	CI 95%	Pr( >|t|)	β	CI 95%	Pr( >|t|)
**Outcome: correct answer**						
PRA	0.27	(− 0.21; 0.75)	0.27	− 0.03	(− 0.57; 0.51)	0.90
AA-Med	**5.73**	**(1.52; 9.93)**	**< 0.01**	2.93	(− 2.12; 7.98)	0.25
AA-Med—squared	**− 0.15**	**(− 0.26; − 0.04)**	**< 0.01**	− 0.08	(− 0.20; 0.047)	0.22
TFA	0.25	(− 0.21; 0.71)	0.29	− 0.04	(− 0.59; 0.52)	0.90
Sex						
Female	Ref			Ref		
Male	− 1.73	(− 6.69; 3.23)	0.49	− 0.87	(− 3.99; 2.26)	0.58
Course grade average	**9.16**	**(7.34; 10.97)**	**< 0.001**	**4.84**	**(2.74; 6.94)**	**< 0.001**
Admission grade	− 1.33	(− 5.13; 2.46)	0.49	− 4.22	(− 8.08; − 0.36)	0.03
Age	− 0.54	(− 2.64; 1.56)	0.61	− 0.23	(− 3.10; 2.64)	0.87
**Outcome: wrong answer**						
PRA	0.19	(− 0.05; 0.44)	0.12	− 0.34	(− 0.78; 0.09)	0.12
AA-Med	1.18	(− 0.99; 3.36)	0.28	**− 0.63**	**(− 1.21; − 0.04)**	**0.04**
AA-Med—squared	− 0.03	(− 0.09; 0.02)	0.25	–	–	–
TFA	0.12	(− 0.12; 0.36)	0.31	0.38	(− 0.07; 0.82)	0.10
Sex						
Female	Ref			Ref		
Male	− 0.84	(− 3.40; 1.73)	0.52	0.71	(− 1.82; 3.23)	0.58
Course grade average	**− 1.75**	**(− 2.69; − 0.81)**	**< 0.001**	− 1.34	(− 3.04; 0.35)	0.12
Admission grade	0.39	(− 1.57; 2.36)	0.69	0.53	(− 2.59; 3.64)	0.74
Age	0.52	(− 0.57; 1.60)	0.35	0.80	(− 1.51; 3.10)	0.50
**Outcome: blank answer**						
PRA	− 0.46	( − 1.08; 0.16)	0.14	0.39	(− 0.46; 1.25)	0.36
AA-Med	**− 6.91**	**(− 12.35; − 1.46)**	**0.01**	0.83	(− 0.32; 1.98)	0.16
AA-Med—squared	**0.18**	**(0.04; 0.32)**	**< 0.01**	–	–	–
TFA	− 0.37	(− 0.96; 0.22)	0.22	− 0.40	(− 1.27; 0.48)	0.37
Sex						
Female	Ref			Ref		
Male	2.57	(− 3.85; 8.99)	0.43	0.20	(− 4.75; 5.15)	0.94
Course grade average	**− 7.40**	**(− 9.75; − 5.06)**	**< 0.001**	**− 3.60**	**(− 6.92; − 0.28)**	**0.03**
Admission grade	0.94	(− 3.98; 5.86)	0.71	3.57	(− 2.54; 9.67)	0.25
Age	0.02	(− 2.70; 2.75)	0.99	− 0.37	(− 4.89; 4.15)	0.87

Pearson risk attitude (PRA), Ambiguity aversion in medicine (AA-Med), and of tolerance for ambiguity (TFA), respectively

Items in **bold** reached statistical significance

Once again, in the Immunology examination, the scale AA-Med showed quadratic association with the number of correct and blank answers, but not with the number of wrong answers (Table 3). The Microbiology examination only showed significant linear association with the number of wrong answers (Table 3).

Having a high course grade average was shown to be associated with a statistically significant increase in the number of correct answers and decrease in the number of blank answers in both examinations, and a decrease in the number of wrong answers only in the Immunology examination.

There was no crude or adjusted association between age, gender, or admission grade and the number of correct, wrong, or blank answers in either examination (Table 3).

## Discussion and conclusion

### Discussion

This study, to our knowledge, the first to investigate the effect of attitude towards ambiguity in students’ response patterns, showed that students with a very high ambiguity aversion in medicine left more answers blank and answered fewer questions correctly, as predicted, in the Immunology exam. However, the same happened to those with very low levels, meaning that an intermediate level of aversion to ambiguity is more advantageous. This means that there is some truth to the saying “the middle path is the way to wisdom” and suggests that students with extreme levels of ambiguity aversion in medicine, with either absolute trust or distrust for ambiguous medical tests or treatments, have more difficulty answering questions for which they only have partial knowledge, and are therefore being penalised in MCEs with formula scoring. However, the same result was not observed in the Microbiology examination, possibly due to its difficulty and discrimination indexes, which were both lower than those recommended (Mahjabeen et al., 2018) and those in previous Microbiology examinations.

The notion of ambiguity is inherent not only to MCEs, as questions and answers could have multiple interpretations, but also to medical practice because of limitations in professional knowledge, uncertainty of diagnosis, therapy, and outcomes as well as patient response unpredictability. Hence, assessing, educating, and even selecting medical trainees regarding and based on their ambiguity tolerance is essential (Geller et al., 1990).

In this study we did not find any statistically significant associations between the students’ Risk Attitude and their response patterns, in accordance with the results from Ndu et al. (2016), neither with their tolerance for ambiguity. This suggests that medical students’ tolerance for risk and ambiguity in their daily life does not affect their answers in medical exams, but their ambiguity aversion in medicine does. This may be due to the fact that the AA-Med scale was the only one specific to Medicine. Since it has been reported that AA-Med scores decreases during medical school (Han et al., 2015), and these students were still in their pre-clinical years, it is possible that in the future they will be better at making decisions in situations in which they do not have complete knowledge, which will be a common occurrence in their daily practice, assuming that the levels also do not become extremely low.

Having a high course grade average was associated with a statistically significant increase in the number of correct answers and decrease in the number of wrong and blank answers in the Immunology exam. This makes sense as better students which had higher grades in the past have a higher probability of answering more questions correctly in future exams. Once again, however, the results were not as clear with in the Microbiology test, where the association between course grade average and number of wrong answers was non-significant.

Furthermore, we did not find any statistically significant associations between the students’ gender and their response patterns, in line with the results from Ndu et al. (2016). It is possible that this was due to our sample’s high female to male ratio with a consequent low power of discrimination. It is also possible that male medical students have a psychological profile when it comes to attitude towards risk and ambiguity closer to the female gender’s and are therefore not significantly different. Hence, they could not be considered to be representative of the entire male population.

To our knowledge, this study was the first of its kind in Portugal, hence our need to translate the scales to Portuguese. However, these scales have not been standardised to the Portuguese population, therefore there is no guarantee that the acuity of the scale was not decreased by the translation, although the fact that the back translation to English showed similar results and that the scales reliability were high suggests that the scales were not heavily affected by the translation.

Another limitation of the study is the use of data collected at two different times, with an interval of about four months between the examinations and the questionnaire: although it is unlikely that there would be very significant personality changes in this period of time, it would be preferable if this period could be reduced. However, it would be important to maintain the order in which information was collected: if students had been asked to complete the questionnaire before taking the examinations, their knowledge of the purpose of the investigation could inappropriately influence their response pattern in the examinations, introducing another bias.

The fact that both examinations included not only the True or False questions which were used to assess the association in the scales, but also other questions without formula scoring, some of which were rated several times higher, could also be a limitation. It might explain why no statistically significant association was found between risk tolerance and the number of questions left blank since students which would normally spend time trying to answer questions for which they only have partial knowledge, might have instead decided to simply leave them blank and spend their extra time answering the remaining questions without formula scoring more carefully, especially since both groups of True or False questions had a low difficulty index.

Finally, the relationship between tolerance of uncertainty and the response pattern in MCEs with formula scoring was only tested on a limited set of True/False questions from two examinations of the second academic year of MIMed, carried out by students from one of the Portuguese medical schools (FMUP). It is possible that these questions were not complex or ambiguous enough to show association with the scales.

## Conclusion

In the future, it would be important to replicate this study with larger samples, including students from different medical schools and course units from several academic years, ensuring that examinations with a larger spectrum of complex and ambiguous questions are used. It would be ideal for the tests under study to contain the same type of MCQs, some with formula scoring and others with number-right scoring, focusing on the same set of knowledge so that direct comparisons could be made with greater certainty. Furthermore, it would be useful to collect this data over a long period of time (i.e., in their first and final years of medical school) to see how the students’ score and response pattern change over time. Finally, we advise the measurement of not only the student’s attitudes toward risk and ambiguity, but also their personality traits, using, for example, the five-factor model.

On a more practical note, we recommend the planning and conduct of seminars for students and teachers about the implications of students’ attitude towards ambiguity in their response patterns in MCEs with formula scoring. The objectives would be to encourage students to become aware of how these characteristics influence the way they respond in this type of examinations, since it has been shown that aversion to ambiguity in medicine can change over time (Han et al., 2015) and, on the other hand, for teachers to (re) equate, based on available scientific evidence, the use of the formula scoring quotation method in future tests, and the use of ambiguous questions which benefit some students over others.

Based on our review of the literature and on this study’s findings, we discourage the use of formula scoring because: it decreases the validity of examinations, since the final result can be influenced by factors extrinsic to the knowledge of students (like their ambiguity aversion in medicine); and because it discourages the use of partial knowledge, which differs from the real environment of clinical practice where physicians frequently have to make decisions based on only partially complete information (Muijtjens et al., 1999). Another argument in favour of number-right scoring is the fact that since the evaluation of students during medical school involves many different examinations, formula scoring’s higher reliability is not very significant since reliability increases when more tests are involved. However, systematic errors that repeatedly penalize some students based on their attitudes or personality traits would not decrease across more measurements.

### Supplementary Information

Below is the link to the electronic supplementary material.

Supplementary file1 (PDF 740 kb)

Supplementary file2 (DOCX 13 kb)

## Data Availability

The datasets used and/or analysed during the current study are available from the corresponding author on reasonable request.

## Abbreviations

MCEs: Multiple choice exams
MCQs: Multiple choice questions
PRA: Pearson risk attitude
AA-Med: Ambiguity aversion in medicine
TFA: Tolerance for ambiguity
MIMed: Integrated master of medicine
