# Supplementary material for: The effect of the attitude towards risk/ambiguity on examination grades: cross-sectional study in a Portuguese medical school
Source: Adv Health Sci Educ Theory Pract. 2024 Jan 15;29(4):1309–21. doi: 10.1007/s10459-023-10305-z (PMC11368994; doi:10.1007/s10459-023-10305-z)
Supplement: Supplementary file 1 — Supplementary file1 (PDF 740 kb) [file 10459_2023_10305_MOESM1_ESM.pdf]

U.PORTO
FMUP FACULDADE DE MEDICINA
UNIVERSIDADE DO PORTO

Questionário sobre risco/ambiguidade

(Não Preencher)

O presente questionário autoaplicado pretende estudar a relação entre as atitudes dos estudantes da FMUP face ao risco e à ambiguidade e os padrões de respostas (número de respostas branco, incorretas e corretas) em exames de escolha múltipla com penalização. O questionário é constituído por 4 secções de perguntas fechadas. O preenchimento do mesmo não deverá demorar mais de 5 minutos. Todas as informações recolhidas neste questionário são confidenciais e serão exclusivamente usadas para o propósito do estudo.

Muito obrigado pela sua colaboração.

Face ao seu processo de tratamento (leitura óptica), este inquérito deve ser preenchido utilizando caneta ou esferográfica preta ou azul e preenchido como mostra o exemplo.
Se eventualmente se enganar a assinalar a sua resposta, deverá colocar uma cruz e preencher o círculo correspondente à resposta que pretende.

Preencha	
assim	●
assim não	⌀ O
anular	⊗ ●

Secção I

Número mecanográfico: NMECANO

Turma: Turma   Sexo: O Masculino O Feminino Sexo   Idade: Idade

Média do curso: Medcurso ,   Média de entrada: , Medentrada

Tem alguma Unidade Curricular em atraso? O Não O Sim atrasoUC   Se **sim**, indique quantas: atrasoUCquantas

Costuma deixar respostas em branco num teste escolha múltipla com descontos (penalização)?

O Nunca O Raramente O Às vezes O Muitas vezes O Sempre brancos

Secção II

De seguida, gostaríamos que respondesse a um conjunto de perguntas sobre **Atitude perante o risco**.

		Discordo Totalmente					Concordo Totalmente
R1	1.Gosto de arriscar.	O 1	O 2	O 3	O 4	O 5	O 6
R2	2.Tento evitar situações com resultados incertos.	O 1	O 2	O 3	O 4	O 5	O 6
R3	3. Arriscar não me incomoda se os ganhos envolvidos forem elevados.	O 1	O 2	O 3	O 4	O 5	O 6
R4	4. Considero a segurança um elemento importante em todos os aspetos da minha vida.	O 1	O 2	O 3	O 4	O 5	O 6
R5	5. Já me disseram que pareço gostar de arriscar.	O 1	O 2	O 3	O 4	O 5	O 6
R6	6. Raramente arrisco quando há outra alternativa.	O 1	O 2	O 3	O 4	O 5	O 6

Secção III

De seguida, gostaríamos que respondesse a um conjunto de perguntas sobre **Aversão à Ambiguidade na Medicina**.

		Discordo Totalmente					Concordo Totalmente
A1	1. Não teria confiança num exame ou tratamento médico, caso os especialistas tivessem opiniões contraditórias acerca do mesmo.	O 1	O 2	O 3	O 4	O 5	O 6
A2	2. Opiniões contraditórias de especialistas acerca de um exame ou tratamento médico deixar-me-iam aborrecido.	O 1	O 2	O 3	O 4	O 5	O 6
A3	3. Não ficaria receoso em experimentar um exame ou tratamento médico, mesmo se os especialistas tivessem opiniões contraditórias sobre este.	O 1	O 2	O 3	O 4	O 5	O 6
A4	4. Se os especialistas tivessem opiniões contraditórias acerca de um exame ou tratamento médico ainda estaria disposto a experimentá-lo.	O 1	O 2	O 3	O 4	O 5	O 6
A5	5. Evitaria tomar uma decisão acerca de um exame ou tratamento médico, se os especialistas tivessem opiniões contraditórias sobre este.	O 1	O 2	O 3	O 4	O 5	O 6

5827

v.s.f.f.

Secção III

De seguida, gostaríamos que respondesse a um conjunto de perguntas sobre **Tolerância para a Ambiguidade**.

		Discordo Totalmente					Concordo Totalmente
T1	1. Fico bastante perturbado quando não sou capaz de seguir a linha de pensamento de outra pessoa.	O 1	O 2	O 3	O 4	O 5	O 6
T2	2. Fico muito ansioso se estiver incerto quanto às responsabilidades envolvidas numa tarefa, em particular.	O 1	O 2	O 3	O 4	O 5	O 6
T3	3. Antes de qualquer tarefa importante, preciso de saber quanto tempo é que esta vai demorar.	O 1	O 2	O 3	O 4	O 5	O 6
T4	4. Não gosto de trabalhar num problema, a não ser que haja possibilidade de obter uma resposta clara e não ambígua.	O 1	O 2	O 3	O 4	O 5	O 6
T5	5. A melhor parte de trabalhar num quebra-cabeças é encaixar aquela última peça.	O 1	O 2	O 3	O 4	O 5	O 6
T6	6. A não ser que sinta que consigo perceber o seu comportamento, estou frequentemente desconfortável com pessoas.	O 1	O 2	O 3	O 4	O 5	O 6
T7	7. Uma boa tarefa é aquela em que o que tem que ser feito e como se faz estão bem definidos.	O 1	O 2	O 3	O 4	O 5	O 6

5827
