# Supplementary material for: The effect of the attitude towards risk/ambiguity on examination grades: cross-sectional study in a Portuguese medical school
Source: Adv Health Sci Educ Theory Pract. 2024 Jan 15;29(4):1309–21. doi: 10.1007/s10459-023-10305-z (PMC11368994; doi:10.1007/s10459-023-10305-z)
Supplement: Supplementary file 2 — Supplementary file2 (DOCX 13 kb) [file 10459_2023_10305_MOESM2_ESM.docx]

Additional File 2

Table 4. Analysis of main components of each scale.

| Scale | Mean (SD) | Cronbach’s Alpha (CI 95%) | % Explained by first component | Range absolute factor loadings  (min-max) |
| --- | --- | --- | --- | --- |
| PRA | 19.41 (4.92) | 0.79 (0.74; 0.83) | 49% | 0.58-0.75 |
| AA-Med | 19.19 (3.86) | 0.69 (0.62; 0.75) | 45% | 0.57-0.76 |
| TFA | 26.48 (5.26) | 0.70 (0.63; 0.76) | 38% | 0.35-0.71 |

Pearson Risk Attitude (PRA), Ambiguity Aversion in Medicine (AA-Med), and of Tolerance For Ambiguity (TFA), respectively
